# Beyond the Joints: Rheumatoid Meningitis as a Neurological Manifestation of Rheumatoid Arthritis

**DOI:** 10.7759/cureus.80764

**Published:** 2025-03-18

**Authors:** Debora Gonzalez, Miguel A Gonzalez Casas, Martin H Gómez Martínez, Stephanie S Tlali Díaz

**Affiliations:** 1 Internal Medicine, Regional General Hospital 1, Mexican Social Security Institute, Queretaro, MEX; 2 Neurology, Institute for Social Security and Services for State Workers, Saltillo, MEX; 3 Neurosurgery, Institute for Social Security and Services for State Workers, Saltillo, MEX; 4 Geriatrics, Regional General Hospital 1, Mexican Social Security Institute, Queretaro, MEX

**Keywords:** autoimmune, central nervous system, leptomeninges, rheumatoid arthritis, rheumatoid meningitis

## Abstract

Rheumatoid meningitis (RM) is a rare and severe extra-articular neurological manifestation of rheumatoid arthritis. Headache is one of the most common symptoms along with focal neurological deficits, neuroinfective symptoms, including seizures, and altered mental status are prevalent. We present a case of a 58-year-old Mexican woman with a history of rheumatoid arthritis since 2018, with positive cyclic citrullinated peptide antibodies and rheumatoid factor. Her clinical presentation began with a right-sided hemicranial headache, which was followed by an acute confusional state and discrete left-sided hemiparesis. The first magnetic resonance imaging (MRI) of the brain revealed meningeal enhancement throughout the right cerebral hemisphere. Cerebrospinal fluid (CSF) analysis showed hyperproteinorrhachia, pleocytosis, and hypoglycorrhachia. A biopsy ruled out infectious and neoplastic processes, leading to a diagnosis of rheumatoid meningitis (RM). This study represents the diagnostic challenge posed by rheumatoid meningitis in the presence of unspecific neurological symptoms due to its similarity with other etiologies, which may delay adequate treatment. It also highlights the usefulness of diagnostic tools that can guide physicians in the early recognition of this pathology, improving the patient's prognosis.

## Introduction

Rheumatoid meningitis is an extra-articular neurological manifestation of rheumatoid arthritis (RA) that is extremely rare and difficult to recognize clinically. In this pathology, the meninges that envelop the brain and spinal cord are affected, leading to inflammation of the dura mater (pachymeningitis) or of the arachnoid and pia mater (leptomeningitis). Although it is usually associated with seropositive and long-standing rheumatoid arthritis, it is not exclusive to this type of disease and can be found in patients with inactive RA and in the early stages of the disease. The clinical presentation of rheumatoid meningitis (RM) is variable and depends on the site of meningeal involvement, which may be focal or diffuse. Symptoms are often nonspecific, including headache, neurological deterioration, seizures, and altered mental status, sometimes mimicking cerebrovascular events or other diagnostic entities [1]. In this study, we report the clinical case of a Latina woman, highlighting the importance of diagnostic suspicion in patients with a history of rheumatoid arthritis who present neurological symptoms. Recognizing this condition is crucial to avoiding misdiagnosis and inappropriate treatments. In this case, an optimal response to steroids was obtained.

## Case presentation

We present the case of a 58-year-old Mexican woman with a relevant family history of a brother diagnosed with stomach cancer. Her significant personal medical history includes a diagnosis of rheumatoid arthritis in 2018, for which she is being treated with leflunomide 20 mg/day and methotrexate 10 mg/week. She also has positive cyclic citrullinated peptide antibodies at a titer of 121.8 U/mL (<5 U/mL) and a positive rheumatoid factor. She has had osteopenia since 2019, which is being treated with calcium and vitamin D. Additionally, she has had migraines since 2019, which are managed with symptomatic treatment. She is also allergic to penicillin.

Her clinical presentation began in 2024 with a right-sided hemicranial headache, which the patient considered migraine, using the usual management based on acetylsalicylic acid, paracetamol, and caffeine. As the headache intensified, she sought medical evaluation and was admitted to the neurology department, where she developed an acute confusional state and mild left-sided hemiparesis. Physical examination showed discrete changes in metacarpophalangeal and interphalangeal joints, with Bouchard nodules, they correspond to chronic changes without inflammation or activity in the joints. Neurological evaluation highlighted disorientation in time and space, as well as abulia and apathy. Left-sided hemiparesis was graded 3/5 on the Daniels scale, along with mild neck stiffness, though Kernig and Brudzinski signs were negative.

**Figure 1:**
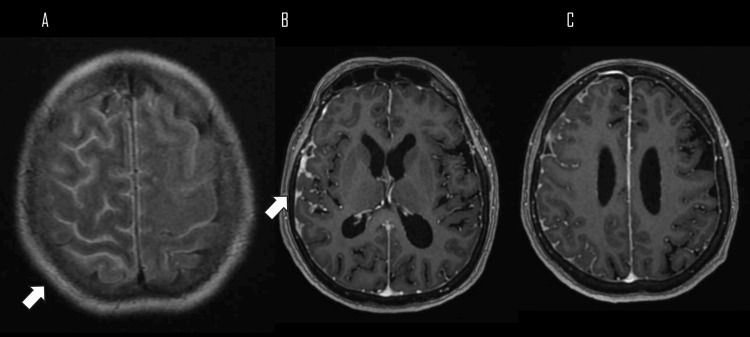
MRI of the brain in T1-weighted contrast-enhanced sequence in axial sections, showing involvement of the leptomeninges of the right cerebral hemisphere. (A) Axial section at the cortical level showing leptomeningeal hyperintensity (arrow) in the frontal lobe and part of the right parietal lobe. (B) Axial section at the level of the anterior horns showing gadolinium enhancement of the leptomeninges in the insular region (arrow). (C) A slide at the level of the ventricular bodies shows increased gadolinium enhancement in the leptomeninges at the frontal lobe, parietal lobe, and midline.

Her initial brain MRI with contrast revealed meningeal enhancement throughout the entire right cerebral hemisphere, predominantly at the supratentorial level, as well as scattered hyperintensities in the white matter in relation to ischemic microangiopathy Fazekas I (Figures 1A-1C), with these findings a total of four lumbar punctures were performed throughout her hospitalization (Table 1).

**Table 1:** Lumbar puncture cytochemical analysis.

Parameter	First puncture	Second puncture	Third puncture	Fourth puncture	Reference interval
Color	Rock water	Rock water	Rock water	Rock water	Rock water
Appearance	Clear	Clear	Clear	Clear	Clear
Glucose	41 mg/dL (50-70 mg/dL)	35 mg/dL (50-70 mg/dL)	57 mg/dL (50-70 mg/dL)	48 mg/dL	50-70 mg/dL
Proteins (Pts)	76 mg/dL	55 mg/dL	32 mg/dL	10 mg/dL	15-45 mg/dL
Erythrocytes	15	0	0	0	Ausent
Leukocytes	17	28	0	0	0-5/mm^3^
Neutrophils	20%	40%	-	-	-
Lymphocytes	80%	60%	-	-	-

Blood tests, including complete blood count, blood chemistry, liver function tests, and electrolytes, were within normal limits. Acute phase reactants were slightly elevated, with CRP at 5 mg/dL and ESR at 30 mm/h. The first cerebrospinal fluid (CSF) analysis showed elevated protein levels (76 mg/dL), pleocytosis (17.6 cells/mm³) predominantly composed of lymphocytes (80%), and hypoglycorrhachia (41 mg/dL). Given the neuroinfection criteria, an etiological investigation was initiated, including the following CSF studies: adenosine deaminase, acid-fast bacilli (AFB) stain, mycobacterial culture, GeneXpert PCR, cryptococcus, KOH preparation, fungal culture, and cytology for malignant cells, all of which were negative. Additionally, immune-related causes were ruled out, with negative anti-N-methyl-d-aspartate (NMDA) receptor antibodies and IgG4 (Table 2).

**Table 2:** Complementary studies in CSF. NMDA: N-methyl-D-aspartate

Complementary studies	Results	Reference interval
Adenosine deaminase	2.61 U/L	0-9 U/L
CSF culture	Negative	Negative
Gram stain bacterioscopy	Negative	Negative
Acid-fast bacilli (AFB)	Negative	Not detected
India ink (Cryptococcus)	Negative	Negative
KOH test	Negative	Negative
Mycobacterial culture	Negative	Negative
GeneXpert PCR	Negative	Negative
Cytology (first)	Acellular	Negative
Anti-NMDA antibodies (serum)	Negative	Negative
Fungal culture	Negative after 30 days of incubation	Negative
Cytology (second)	Acellular	Negative
IgG subclasses/IgG 4	138 mg/dL (negative)	3-201 mg/dL
KOH test	Negative	Negative
Galactomannan (Aspergillus)	0.04 (negative)	<0.50

**Figure 2:**
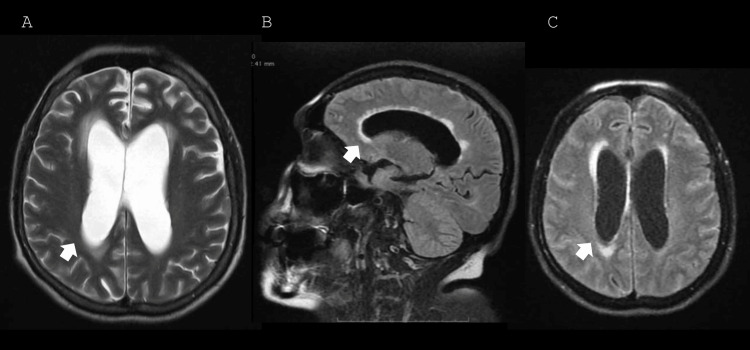
MRI of the patient in T2/FLAIR sequence showing hydrocephalus with transependymal edema. (A) Axial T2-weighted section at the level of the ventricular bodies showing ventricular dilation (arrow) and transependymal leakage, predominantly anterior and posterior. (B) Sagittal FLAIR section at the level of the ventricular bodies showing ventricular dilation and transependymal edema (arrow) extending throughout the entire ventricular body. (C) Axial FLAIR section at the level of the ventricular bodies showing hydrocephalus with transependymal edema (arrow), including extension toward the midline. FLAIR: fluid-attenuated inversion recovery

Days later the patient exhibited a decreased level of consciousness. A second brain MRI showed ventricular system dilation with transependymal leakage (Figures 2A-2C). Given these findings, an endoscopic third ventriculostomy was performed. However, due to the lack of clinical improvement, a ventriculoperitoneal shunt was placed, leading to an improvement in her level of consciousness.

**Figure 3:**
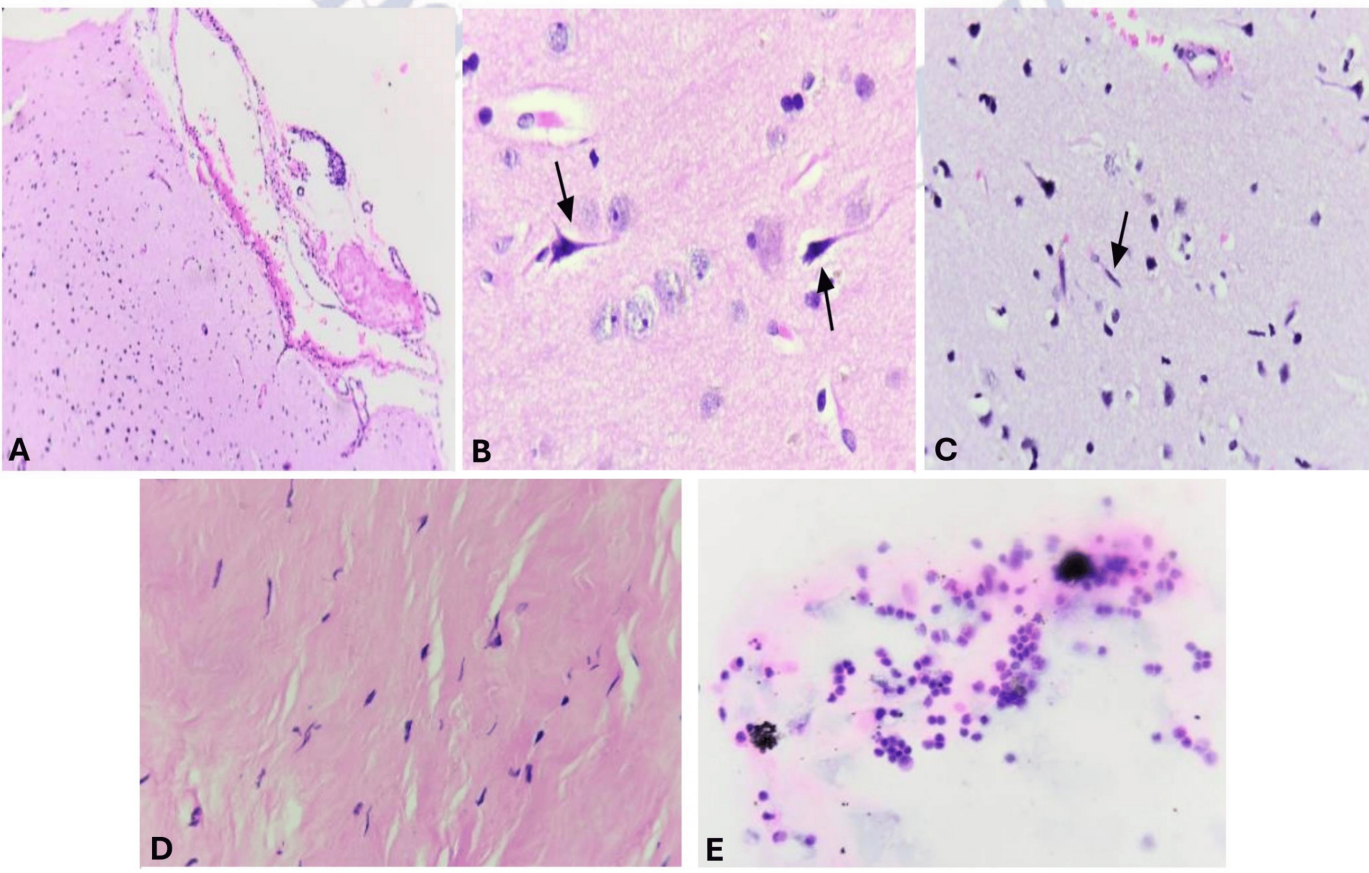
Histological sections stained with hematoxylin and eosin show fragments of the cerebral cortex and white matter. Some atrophic neurons and intracellular pigment accumulation (lipofuscin) are identified. The neuropil is finely fibrillar with the presence of a few activated microglial cells. White matter with fascicular oligodendrocytes is also observed. The leptomeninges exhibit dilated and congested blood vessels. The cerebrospinal fluid cytology shows mononuclear leukocytes and foamy macrophages. (A) Dilation of the leptomeningeal vessels (H&E, 10x). (B) Focal neuronal atrophy (arrows) (H&E, 40x). (C) Activated microglial cells (arrow) (H&E, 40x). (D) Dura mater composed of dense regular collagenous connective tissue with scattered fibroblasts (H&E, 40x). (E) Mononuclear inflammatory cells and foamy macrophages (Papanicolaou, 40x).

As part of the continued etiological study, a brain biopsy of the right temporal lobe and leptomeninges was performed via craniectomy to ensure an adequate biopsy sampling, revealing the following histopathological findings: cortical atrophy with activated microglia, dilated and congested blood vessels in the leptomeninges, mononuclear leukocytes, and foamy macrophages. No neoplastic cells were identified. There was no histological evidence of an infectious or neoplastic process in the examined tissue (Figures 3A-3E). Based on the brain biopsy findings, infectious and neoplastic processes were ruled out, leading to the conclusion that this was a complication of her underlying rheumatologic disease, known as rheumatoid meningitis.

**Figure 4:**
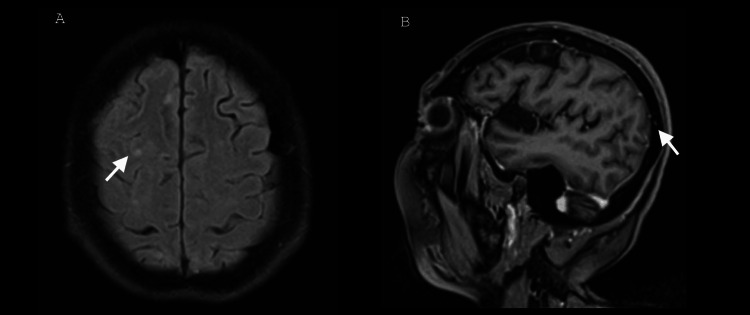
MRI of the patient in FLAIR and T1-weighted contrast-enhanced sequences, post-treatment. (A) Axial section at the cortical level of the frontoparietal lobe showing resolution of meningeal enhancement, with only residual ischemic microangiopathy (arrow). (B) Sagittal T1-weighted contrast-enhanced section showing absence of gadolinium uptake in the leptomeninges (arrow) of the right cerebral hemisphere. FLAIR: fluid-attenuated inversion recovery

Management was administered with intravenous steroids, consisting of five pulses of 1 g of methylprednisolone, resulting in clinical improvement with resolution of headache, motor deficit, and cognitive alterations. A simple and contrast-enhanced brain magnetic resonance imaging (MRI) study showed resolution of meningeal enhancement in the right cerebral hemisphere, previously observed in prior MRIs, along with normalization of cerebrospinal fluid (CSF) cytochemical values in the follow-up analysis (Figures 4A, 4B). Upon hospital discharge, the patient continued disease-modifying treatment for rheumatoid arthritis, without complications or neurological relapses during follow-up.

## Discussion

Central nervous system (CNS) involvement in rheumatoid arthritis (RA) is among the least common and most challenging to recognize clinically. Rheumatoid meningitis (RM) is a rare and severe manifestation of rheumatoid arthritis. Typically, the cerebral meninges are usually more affected than the spinal meninges, leading to pachymeningitis and/or leptomeningitis [1].

The prevalence of this complication remains unknown due to a lack of extensive research on the subject [2]. This patient initially presented with a headache, which worsened despite symptomatic treatment. Headache is one of the most common symptoms, along with focal neurological deficits. However, the clinical presentation of this condition varies depending on the affected meninges, whether diffuse or patchy. Symptoms of neuroinfection are predominant, including seizures, altered mental status, cranial nerve involvement, stroke-like symptoms, encephalopathy, hemiparesis, or paraparesis, although these occur less frequently [1,3].

Diagnosis is largely supported by imaging. Brain magnetic resonance imaging (MRI) typically reveals meningeal thickening, which may be focal or diffuse. Leptomeningeal enhancement with gadolinium is the most common finding, followed by pachymeningeal enhancement. Other observed findings include edema, ventricular dilation, and optic nerve enhancement [4,5].

Cerebrospinal fluid (CSF) analysis typically shows pleocytosis with a predominance of lymphocytes, along with a moderate protein elevation, although CSF findings may be normal. The primary purpose of lumbar puncture is to rule out infectious processes as part of the diagnostic approach. In the present case, most infections, including common causes such as tuberculosis, fungal and parasitic infections, and other autoimmune processes, were excluded. This is essential since rheumatoid meningitis is a diagnosis of exclusion [6,7].

After a literature review, the MRI findings of unilateral meningeal involvement, along with the CSF results, aligned with those reported in patients with rheumatoid meningitis. Following an extensive exclusion of infectious and neoplastic processes, our patient was diagnosed with rheumatoid meningitis.

Histopathological evidence is necessary to establish the diagnosis, although pathological findings are often nonspecific. In the present patient, a biopsy was performed simultaneously with decompressive surgery due to the cerebral edema she had developed. Three abnormal histological patterns have been identified, which include rheumatoid nodules, the most specific finding for rheumatoid meningitis (RM) [8]. These are histologically identical to subcutaneous rheumatoid nodules; however, they are more frequently found in autopsy cases and less commonly in biopsy samples. Nonspecific meningeal inflammatory changes, such as mononuclear cell infiltration, lymphocytic infiltration, and plasma cells, are also observed. Finally, vasculitis is present in a minority of cases [9,10].

Treatment includes the use of glucocorticoids, initially administered as intravenous methylprednisolone pulses, either alone or in combination with disease-modifying drugs, azathioprine, or followed by cyclophosphamide. Successful outcomes have also been reported with rituximab, an anti-CD20 monoclonal antibody. Although treatment can resolve the acute phase, the possibility of relapse cannot be ruled out. Despite intensive treatment, rheumatoid meningitis remains associated with a high mortality rate due to the disease itself and its complications [11,12].

## Conclusions

In conclusion, rheumatoid meningitis continues to be a diagnosis of exclusion and is challenging to recognize since there are no diagnostic criteria for rheumatoid meningitis (RM) so far. Considering that the pathology is mostly restricted to focal damage to the meninges and cortex, clinical manifestations tend to be nonspecific and can mimic another neuroinfectious process. It should be considered as a differential diagnosis in the context of a patient with rheumatoid arthritis in conjunction with imaging findings of unilateral meningeal involvement. It is necessary to exclude other infectious, neoplastic, vasculitis, or other autoimmune causes.

However, according to cases described in the literature, although seropositive rheumatoid arthritis patients are more susceptible to developing this condition, rheumatoid meningitis does not always present alongside clinically evident rheumatoid arthritis. As this is a rare clinical case, the observations made cannot be extrapolated to all patients with rheumatoid arthritis who develop neurological manifestations. Likewise, the absence of long-term follow-up limits the possibility of evaluating the sustained clinical evolution, the definitive response to immunosuppressive treatment, and the risk of recurrence. Finally, given the rarity of rheumatoid meningitis, the limited experience in its management makes it difficult to establish firm conclusions about the most appropriate therapeutic approach. This highlights the need to develop diagnostic guidelines as well as standardized treatment protocols.
